# Synthesis of the Marine Pyrroloiminoquinone Alkaloids, Discorhabdins

**DOI:** 10.3390/md8041394

**Published:** 2010-04-21

**Authors:** Yasufumi Wada, Hiromichi Fujioka, Yasuyuki Kita

**Affiliations:** 1 Graduate School of Pharmaceutical Sciences, Osaka University, 1-6 Yamada-oka, Suita, Osaka, 565-0871, Japan; 2 College of Pharmaceutical Sciences, Ritsumeikan University, 1-1-1 Nojihigashi, Kusatsu, Shiga, 525-8577, Japan

**Keywords:** discorhabdin alkaloid, marine sponge, cytotoxic activity

## Abstract

Many natural products with biologically interesting structures have been isolated from marine animals and plants such as sponges, corals, worms, *etc*. Some of them are discorhabdin alkaloids. The discorhabdin alkaloids (discorhabdin A-X), isolated from marine sponges, have a unique structure with azacarbocyclic spirocyclohexanone and pyrroloiminoquinone units. Due to their prominent potent antitumor activity, discorhabdins have attracted considerable attention. Many studies have been reported toward the synthesis of discorhabdins. We have accomplished the first total synthesis of discorhabdin A (**1**), having the strongest activity *in vitro* among discorhabdins in 2003. In 2009, we have also accomplished the first total synthesis of prianosin B (**2**), having the 16,17-dehydropyrroloiminoquinone moiety, by a novel dehydrogenation reaction with a catalytic amount of NaN_3_. These synthetic studies, as well as syntheses of the discorhabdins by various chemists to-date, are reviewed here.

## 1. Introduction

Marine organisms contain various elements that are important for their survival and for their relationships with other living bodies. The nature, action and role of many of these elements have not yet been elucidated. Many marine organic compounds have been shown to be also useful for humans; as drugs, agricultural medicines, cosmetics and health foods [1–5]. In particular, a number of marine drugs have been developed in recent years. Marine natural products offer challenging targets to synthetic chemists due to their complicated structures that exhibit remarkable biological activities [6–11]. However, the chemical synthesis of marine drugs is of upmost importance as a synthetic supply is needed when it is difficult to maintain the amount of the natural source.

**Figure 1:**
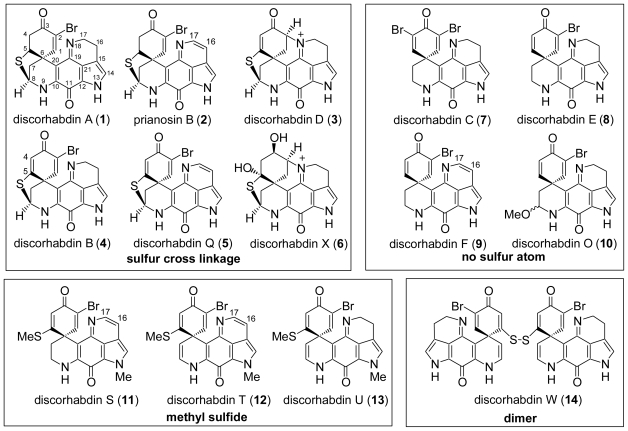
The structure of discorhabdin alkaloids.

In the past several years, many new pyrroloiminoquinone alkaloids have been isolated from marine sources. The pyrroloiminoquinone alkaloid family consists of discorhabdins [12–16], prianosins [17], makaluvamines [18–21], batzellins [22–23], wakayins [24] and damirones [25–26], and have potent antitumor activities derived from their unique fused ring structures [27]. Among them, the discorhabdin alkaloids were isolated from marine sponges such as New Zealand sponges of the genus *Latrunculia*, the Okinawan sponge *Prianos melanos*, the Fijian sponge *Zyzzya cf. Marsailis*, *etc.* [12–16]. All of them have unique structures, with azacarbocyclic spirocyclohexanone and pyrroloiminoquinone structures. Figure 1 shows some representative discorhabdin alkaloids. Among the various isolated discorhabdins (A–X), discorhabdins A (**1**) [28,29], B (**4**) [28,29], D (**3**) [30], H [15], I [14], J [12], L [14], M [12], N [12], Q (**5**) [31], R [32], X (**6**) [33], and prianosins B (**2**), D [34] have a sulfur-containing fused ring structure. Discorhabdins S (**11**), T (**12**) and U (**13**) [13] have a methyl sulfide moiety. Discorhabdin W (**14**) [35] is a dimeric structure with a disulfide bond, while the others have no sulfur atom. Furthermore, discorhabdins F (**9**) [36], Q, S, T and prianosin B (**2**) contain a 16,17-dehydropyrroloiminoquinone moiety. Because of their prominent potent antitumor activity and unusual ring structure, pyrroloiminoquinone alkaloid synthesis has attracted the interest of many organic chemists, and over the last decade, the total synthesis of a few of them as well as synthetic approaches has appeared. Our group accomplished the first total syntheses of discorhabdin C (**7**) [37], makaluvamine F [38,39] and discorhabdin A (**1**) [40–43]. In 2009, we also accomplished the first total synthesis of prianosin B (**2**) [44]. In this review, we describe the total syntheses of the discorhabdins by various chemists to-date.

## 2. Studies of the Total Synthesis of Discorhabdin C

In 1986, discorhabdin C (**7**) was isolated from the sponge *Latrunculia* in New Zealand by Munro *et al.* and was found to exhibit a strong cytotoxicity against tumor cells (ED_50_ = 30 ng/mL against P388 and <100 ng/mL against L1210) [45]. Discorhabdin C was the first isolated of the discorhabdins and much attention has been paid to the total synthesis of this attractive target.

### 2.1. Our Synthesis [37]

**Scheme 1:**
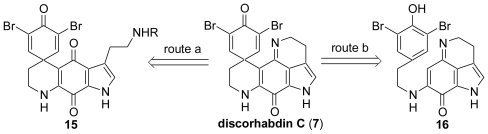
Retrosynthetic analysis for discorhabdin C.

The problems in the synthesis of discorhabdin C are the construction of highly fused ring systems and the formation of the indoloquinone imine moiety. An overview of our approaches to the synthesis of discorhabdin C is illustrated in Scheme 1. One approach involves the imine formation between the tryptamine amine of the side chain and the indoloquinone carbonyl as the final step of the synthesis (route a). Another approach involves the oxidative coupling of the indoloquinone imine by phenyliodine bistrifluoroacetate (PIFA) as the final step (route b) [46].

**Scheme 2:**
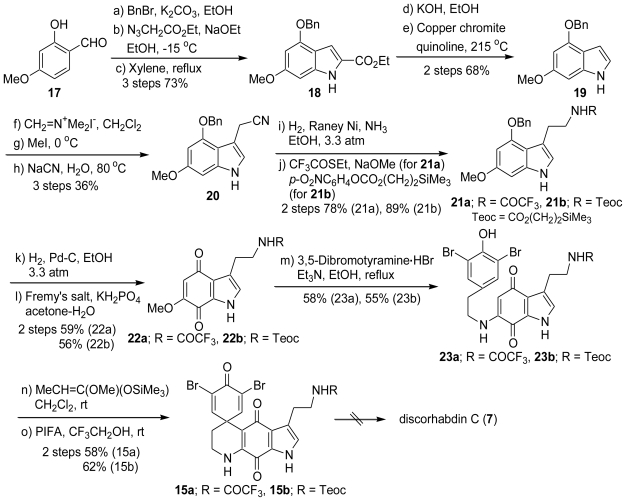
The synthetic approach toward discorhabdin C *via* route a.

A reasonable starting material for route a is 2-hydroxy-4-methoxybenzaldehyde (**17**). The benzylation of **17** with BnBr and K_2_CO_3_ followed by condensation with ethyl azidoacetate (N_3_CH_2_CO_2_Et) in ethanolic sodium ethoxide gave the vinyl azide, which was decomposed in boiling xylene to give the 2-(ethoxycarbonyl)indole (**18**) in 73% yield over three steps. Hydrolysis of the ester group of **18** with KOH gave indolecarboxylic acid, which was decarboxylated with copper chromite under thermal conditions to give the 4,6-disubstituted indole (**19**). The treatment of the indole **19** with dimethyl(methylene)ammonium iodide (CH_2_=N^+^Me_2_I^−^) gave the 3-(dimethylamino)methyl derivative. The dimethylamino group was replaced by a cyano group using NaCN *via* the quaternary salt to yield the 3-(cyanomethyl)indole (**20**). Catalytic hydrogenation of the cyano group of **20** with Raney Ni followed by protection of the resulting amino group with the trifluoroacetyl or the [(trimethylsilyl)ethoxy]carbonyl (Teoc) groups afforded **21a** (R = COCF_3_) and **21b** (R = Teoc), respectively. Debenzylation of **21a**,**b** with reduction followed by oxidation using Fremy’s salt gave the corresponding quinones (**22a**,**b**). The treatment of **22a**,**b** with 3,5-debromotyramine hydrobromide gave the phenol derivatives (**23a**,**b**). Silylation of **23a**,**b** with *o*-silylated ketene acetal (MeCH=C(OMe)(OSiMe_3_)) followed by oxidation with PIFA resulted in the desired intermediate (**15a**,**b**). Unfortunately, all attempts to effect the final imine formation between the tryptamine nitrogen and the indoloquinone carbonyl in these types of intermediates failed (Scheme 2).

**Scheme 3:**
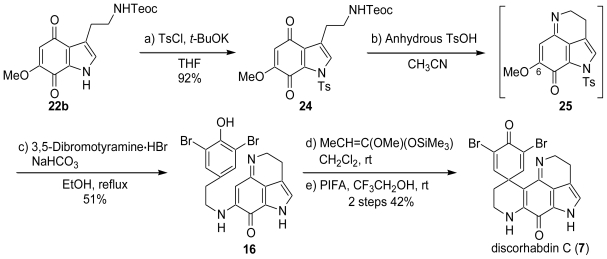
The total synthesis of discorhabdin C *via* route b.

An alternative approach (route b), in which phenolic coupling of the previously produced aminoindoloquinone imine (**16**) is employed in the final step, accomplished the first total synthesis of discorhabdin C (**7**). The direct imine formation from the indoloquinone was achieved by protection of the indoloquinone nitrogen with the tosyl group followed by an acidic dehydrative treatment. The treatment of **22b** with *p*-toluenesulfonyl chloride gave the *N*-tosylate (**24**). Deprotection of the Teoc group of **24** with anhydrous *p*-toluenesulfonic acid in acetonitrile in the presence of 3Å molecular sieves and sodium bicarbonate yielded an unstable indoloquinone imine (**25**), which was subjected to the following one-pot transformation without further purification. When treated with 3,5-dibromotyramine hydrobromide in ethanol, the indoloquinone imine underwent a facile substitution reaction and subsequent detosylation to give the phenolic aminoindoloquinone imine (**16**). The conversion of **16** into its corresponding silylether and the subsequent oxidative coupling reaction using PIFA gave rise to discorhabdin C (**7**) (Scheme 3).

This efficient route was used for the synthesis of a wide variety of previously inaccessible sulfur-containing discorhabdin and prianosin alkaloids (vide infra).

### 2.2. Yamamura’s Synthesis [47,48]

**Scheme 4:**
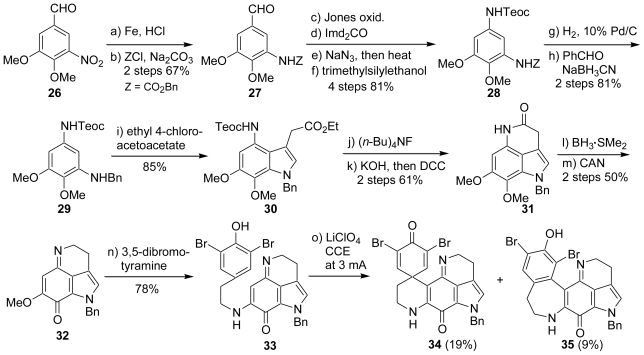
The synthesis of benzyldiscorhabdin C by Yamamura *et al*.

At the same time as our report, Yamamura *et al.* also reported the total synthesis of discorhabdin C (**7**). Their synthesis was initiated from the conversion of the known nitrobenzaldehyde (**26**) into the corresponding amide compound (**27**) in a two-step procedure: reduction of the nitro group followed by Z protection of the generated amine. Manipulation of **27** involving the Curtius reaction provided the diaminobenzene, whose two amino functions could be differentiated by the diverse protection groups (Teoc and Z). Thus, after reductive removal of the benzyloxycarbonyl group, the generated amine was benzylated in the usual manner to give **29**. Among a variety of methods for indole-skeleton constructions, they chose the reaction of **29** with ethyl 4-chloroacetoacetate, which gave rise to the simultaneous introduction of a C_2_ unit at the C-3 position of the indole (**30**). Access to the tetrahydropyrroloquinoline (**32**) was effected by Gannortt’s procedure, *i.e.*, the stepwise removal of the amino and carboxylic acid protective groups of **30** followed by lactam formation provided the tricyclic product (**31**) in a good overall yield. The amide function of **31** was reduced with BH_3_·SMe_2_, and the resulting amine was oxidized with CAN (ceric ammonium nitrate) to produce the expected tetrahydropyrroloquinoline (**32**) in two steps, which further underwent a nucleophilic substitution reaction with 3,5-dibromotyramine to furnish **33** in 78% yield. The electrolysis of bromophenol (**33**) furnished the expected benzyldiscorhabdin C (**34**) accompanied by the ring-expanded product (**35**) (Scheme 4).

**Scheme 5:**
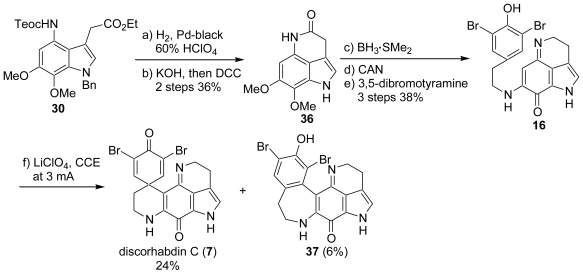
The total synthesis of discorhabdin C by Yamamura *et al*.

Unfortunately, in the last step, all efforts made to remove the benzyl protective group of **34** were unsuccessful. Therefore, they synthesized the bromophenol derivative (**16**). The indole (**30**) was hydrogenated under acidic conditions to remove the benzyl and Teoc protective groups, and the succeeding lactamization as in the case of **30** yielded **36**. The conversion of **36** into **16** smoothly proceeded according to the procedure for the synthesis of **33**. Upon anodic oxidation, **16** produced the desired discorhabdin C (**7**) in 24% yield accompanied by the ring-expanded product (**37**) in 6% yield (Scheme 5).

### 2.3. Heathcock’s Synthesis [49]

**Scheme 6:**
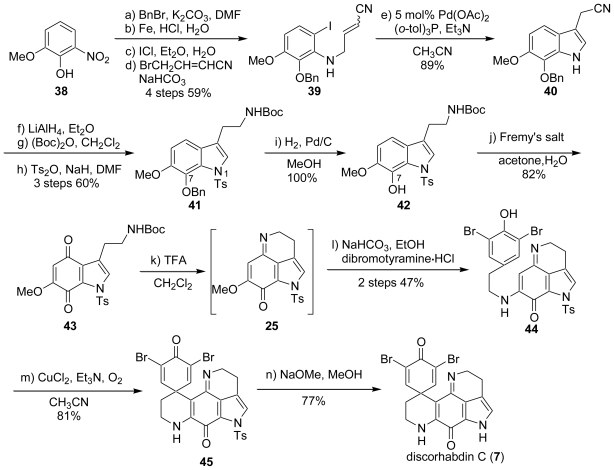
The total synthesis of discorhabdin C by Heathcock *et al*.

In 1999, Heathcock *et al*. accomplished the total synthesis of discorhabdin C (**7**) by a phenolic coupling reaction with cupric chloride. 2-Nitroguaiacol (**38**) was chosen as the starting material. The benzylation of the phenolic hydroxyl group and reduction of the nitro group with Fe and HCl gave the corresponding amino compound, which underwent regioselective iodination with ICl to provide the *ortho*-iodo derivative **39**. The Heck reaction of **39** with Pd(OAc)_2_ and (*o*-tol)_3_P yielded an indole (**40**), which was followed by reduction with LiAlH_4_, and protection with Boc and Ts to provide the 7-benzyloxy-1-tosyl compound (**41**). The debenzylation of **41** with H_2_ and Pd/C afforded the 7-hydroxyindole (**42**), of which treatment with Fremy’s salt gave the *para*-quinone (**43**). The deprotection of **43** with TFA (CF_3_CO_2_H) produced the indoloquinoneimine (**25**). The imine (**25**) underwent a nucleophilic substitution reaction with 3,5-dibromotyramine to furnish **44**. The treatment of **44** with three equivalents of CuCl_2_ and four equivalents of Et_3_N under O_2_ bubbling gave *N*-tosyldiscorhabdin C (**45**), of which the detosylation with NaOMe resulted in the formation of the expected discorhabdin C (**7**) (Scheme 6).

Heathcock *et al.* also achieved the total synthesis of (±)-discorhabdin E (**8**) [49]. The imine (**25**) underwent a nucleophilic substitution reaction with *o*-bromotyramine to furnish **46**. The treatment of **46** with three equivalents of CuCl_2_ and four equivalents of Et_3_N under O_2_ bubbling gave *N*-tosyldiscorhabdin E (**47**), the detosylation of which with NaOMe resulted in the formation of the expected (±)-discorhabdin E (**8**).

## 3. Studies of the Total Synthesis of Sulfur-Containing Makaluvamine, Makaluvamine F (48) [38,39]

**Scheme 8:**
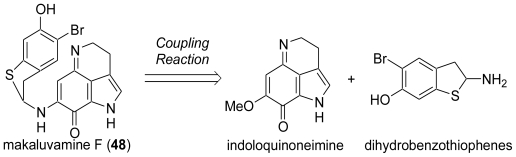
Retrosynthetic analysis of makaluvamine F (**48**).

Among the makaluvamines, makaluvamine F (**48**) has the most potent biological activities (e.g., cytotoxicity towards the human colon tumor cell line HCT-116 (IC_50_ = 0.17 μM) and inhibition of topoisomerase II). Makaluvamine F (**48**) has an α-aminodihydrobenzothiophene skeleton, which is a labile *N,S*-acetal structure present in all sulfur-containing discorhabdins. Synthetic studies of the makaluvamines and discorhabdins have been carried out by several groups. However, in most cases, these efforts have been devoted only to the diverse preparations of the pyrroloiminoquinone and spirodienone units. To the best of our knowledge, the total syntheses of the sulfur-containing discorhabdins and makaluvamine F had not been reported until our synthesis, despite their potent cytotoxicity and their unique structure. This is probably due to the difficulty in construction of the labile and highly strained *N,S*-acetal skeletons. In our efforts to synthesize the discorhabdin alkaloids, we succeeded in the total synthesis of discorhabdin C (**7**) using the PIFA-mediated spirocyclization reaction. Thus, the remaining challenge for the total syntheses of these targets (the sulfur-containing family) was the construction of the labile and highly strained *N,S*-acetal skeletons. Our synthetic strategy for the total synthesis of makaluvamine F (**48**) is shown in Scheme 8.

### 3.1. Facile and Direct Synthesis of the Pyrroloiminoquinone Unit [50]

**Scheme 9:**
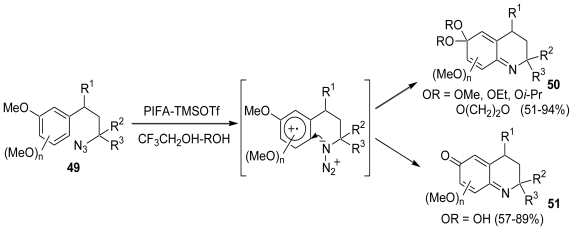
Intramolecular cyclization of phenylethers bearing an alkylazido group.

We found that the treatment of phenyl ethers with an alkylazido group (**49**) in the presence of PIFA-TMSOTf in (CF_3_)_2_CHOH-ROH or CF_3_CH_2_OH-ROH gave the corresponding quinone imine dimethylacetals (**50**), which in (CF_3_)_3_CHOH-H_2_O or CF_3_CH_2_OH-H_2_O gave the corresponding quinine imines (**51**) [51] (Scheme 9). Quinone imines and quinone imine monoacetals have been proposed as the intermediates in a number of biological processes. Quinone imines were also found in the structure of marine alkaloids.

**Scheme 10:**
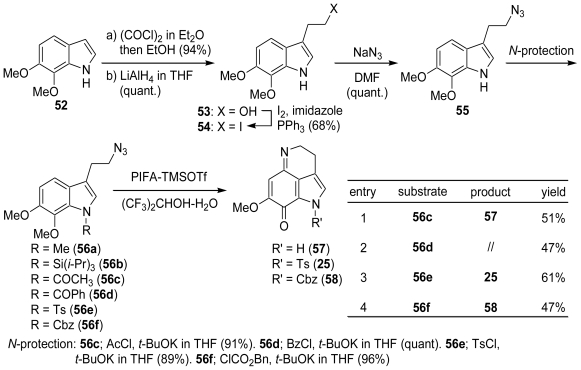
A novel synthetic method for pyrroloiminoquinones.

We have developed a facile and direct preparation of the quinone imine derivatives, and very recently reported the synthesis of pyrroloiminoquinones from the indole bearing an alkylazido group [52]. The starting substrate was prepared as follows. The treatment of 6,7-dimethoxyindole (**52**) with oxalyl chloride followed by LAH reduction gave 3-(hydroxyethyl)indole (**53**) (95% yield), which was converted to 3-(azidoethyl)indole (**55**) *via* 3-(iodoethyl)indole (**54**) in 65% yield. The *N*-protected 3-(azidoethyl)indoles (**56a–f**) were readily prepared from **55** by standard alkylation and acylation methods. First, we examined the reaction of **55** with PIFA-TMSOTf, but only a complex mixture was obtained. The reactions of **56a** (R = Me) and **56b** (R = Si(*i*-Pr)_3_) with PIFA-Me_3_SiOTf also gave complex mixtures, probably due to the side reactions occurring at the 1- or 3-position on the indole nucleus. In fact, several groups have already reported that a 1-, 2- or 3-substituted indole derivative is obtained from the reaction of indoles with iodobenzene diacetate. Therefore, we examined the reactions of the 1-protected indoles (**56c–f**) with PIFA-Me_3_SiOTf in the presence of H_2_O. We found that the corresponding *N*-deprotected pyrroloiminoquinones (**57**) or the *N*-protected pyrroloiminoquinones (**25** and **58**) were obtained in moderate yields when the indoles protected with an electron-withdrawing group, such as the acetyl, benzoyl, tosyl or benzyloxycarbonyl group, were treated with PIFA-Me_3_SiOTf in (CF_3_)_2_CHOH-H_2_O (50:1) (Scheme 10).

### 3.2. Synthesis of the Dihydrothiophene Units [52,53]

**Scheme 11:**
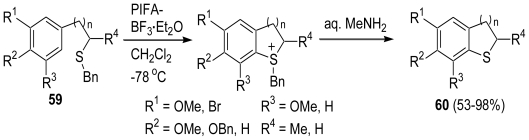
Intramolecular cyclization of phenylethers bearing an alkyl sulfide group.

In our previous studies, we developed the direct sulfenylation of phenyl ethers using PIFA [54,55]. Furthermore, we developed the intramolecular cyclization of phenyl ethers (**59**) bearing an alkylsulfide group using PIFA in (CF_3_)_2_CHOH and PIFA-BF_3_·Et_2_O in CH_2_Cl_2_ that specifically produced the corresponding sulfur-containing heterocycles (**60**) (Scheme 11) [52].

**Scheme 12:**
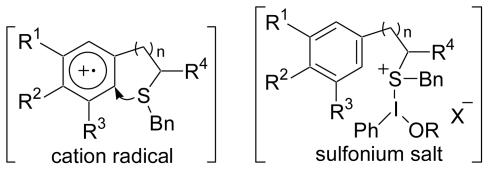
A plausible mechanism for the cyclization reaction of **59**.

A plausible reaction mechanism is that the electron-rich aromatic ring is initially oxidized by the activated PIFA *via* a single electron transfer (SET), and then the sidechain attacks the radical cation. Although other mechanisms, such as the one *via* the sulfonium salt could also be possible, we proposed the mechanism *via* the radical cation (Scheme 12).

**Scheme 13:**
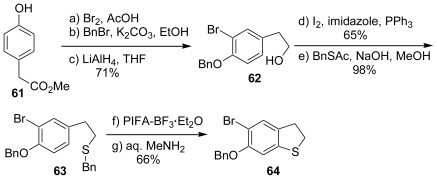
Synthesis of dihydrobenzothiophene.

We constructed the dihydrobenzothiophenes using the direct sulfenylation of phenol ethers with PIFA and arylthiols. The synthesis was initiated from the methyl 4-hydroxyphenylacetate (**61**) to afford the phenol ether (**63**) bearing an alkyl sulfide side chain in five steps. The treatment of the phenol ether (**63**) with PIFA-BF_3_·Et_2_O followed by the treatment of aqueous MeNH_2_ provided the dihydrobenzothiophenes (**64**) (Scheme 13).

**Scheme 14:**
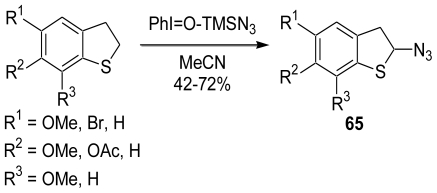
Synthesis of α-azidodihydrobenzothiophene derivatives.

**Scheme 15:**
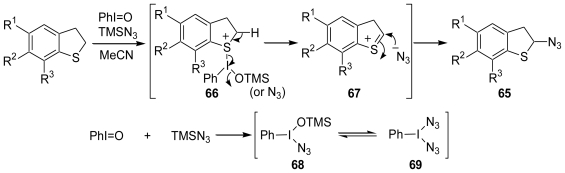
A plausible reaction mechanism for α-azidation of dihydrobenzothiophene derivatives.

Next, we attempted to synthesize the α-azidodihydrobenzothiophenes, which can serve as an *N,S*-acetal equivalent, by the introduction of an azido group to the dihydrobenzothiophene derivatives. After considerable effort, we found a novel and direct α-azidation method for cyclic sulfides using a combination of PhI=O and Me_3_SiN_3_ (Scheme 14) [53]. A plausible reaction mechanism is proposed in Scheme 15. The sulfonium cation (**66**) initially formed by the reaction of the sulfide with PhI=O-Me_3_SiN_3_, whose combination was studied by Magnus and co-workers, is then deprotonated to give a cation intermediate (**67**). The azido anion attacks the α-position of **67** to produce the α-azido sulfide (**65**).

**Scheme 16:**
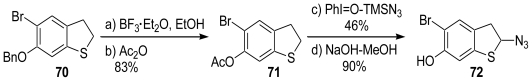
The synthesis of 2-azido-5-bromo-6-hydroxydihydrobenzothiophene (**72**).

Although azidation of 5-bromo-6-benzyloxydihydrobenzothiophene (**70**) gave only a trace amount of the expected α-azido compound, treatment of the acetylated compound (**71**) with PhI=O and Me_3_SiN_3_ followed by hydrolytic deprotection of the 6-acetoxy group provided the 2-azido-5-bromo-6-hydroxydihydrobenzothiophene (**72**) (Scheme 16).

### 3.3. Total Synthesis of (±)-Makaluvamine F (**48**) [38,39]

**Scheme 17:**
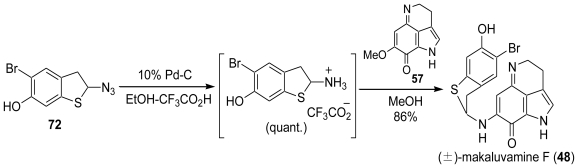
The total synthesis of (±)-makaluvamine F (**48**).

Catalytic hydrogenation of **72** followed by the coupling reaction with **57** in MeOH in the presence of Me_3_SiOTf gave makaluvamine F only in a poor yield. Finally, we found that the catalytic hydrogenation of **72** using 10% Pd-C in the presence of four equivalents of trifluoroacetic acid (TFA) resulted in complete reduction leading to a TFA salt in quantitative yield without any side reactions. The final coupling reaction in MeOH between both synthetic precursors, the TFA salt and **57**, proceeded to give the TFA salt of makaluvamine F (**48**) (Scheme 17).

## 4. Total Synthesis of Discorhabdin A [40–43]

In 1987, prianosin A [28] was isolated from the Okinawan sponge *Prianos melanos* by Kobayashi *et al.*, and in 1988, discorhabdin A [29] was isolated from New Zealand sponges of the genus *Latrunculia* by Munro *et al.* Some years later, it was found that prianosin A and discorhabdin A were the same compound. Discorhabdin A (prianosin A) is antimicrobial and has a strong cytotoxicity (IC_50_ values of 37 ng/mL, 14 ng/mL, 40 ng/mL and 13 ng/mL against L1210, L5178Y, P388 and xrs-6 *in vitro*, respectively, and ED_50_ values of 50 ng/mL against P388). Our next synthetic target was discorhabdin A (**1**), which has a unique sulfur-containing fused ring system incorporating azacarbocyclic spirocyclohexanone and pyrroloiminoquinone systems, and shows the most powerful cytotoxic activity among the isolated discorhabdins.

**Scheme 18:**
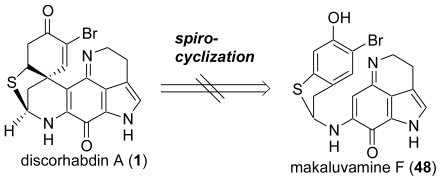
Synthetic approach of discorhabdin A from makaluvamine F.

First, on the basis of the hypothesis by Munro and co-workers [56], we examined the biosynthetically plausible route from makaluvamine F (**48**) using our previously developed oxidative spirocyclization reaction with PIFA. As a result, the oxidative cyclization of makaluvamine F (**48**) as well as the trimethylsilylated makaluvamine F using PIFA under various conditions yielded a complex mixture, probably due to the high reactivity of the iodine(III) reagent toward the sulfide group, the aminoiminoquinone skeleton, and the phenolic OH group in makaluvamine F (**48**) (Scheme 18).

**Scheme 19:**
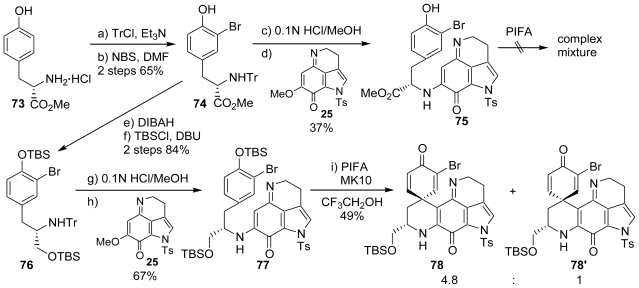
The synthesis of spirodienone.

Therefore, we altered the synthetic strategy. The new strategy included the pre-construction of the spirodienone system using the hypervalent iodine(III) reagent and the final introduction of the sulfur group to the cross-linked system. We first examined the shorter path *via* an amino acid derivative. The tritylation of the l-tyrosine methyl ester hydrochloride (**73**) with TrCl and Et_3_N followed by mono-bromination with NBS yielded **74** (two steps, 65% yield). The coupling reaction of **74** with pyrroloiminoquinone **25**, which was prepared by our previously developed PIFA-induced pyrroloiminoquinone formation, provided **75** as its CF_3_CO_2_H salt (**75**·TFA). We then examined the oxidative spirocyclization reaction of **75**·TFA using PIFA. Although the various reaction conditions tested, this reaction did not give the corresponding spirodienone, but yielded a complex mixture; probably because generation of the free base of **75** was difficult. This might be because **75**·TFA has an active methine proton and readily decomposed under basic conditions (even in the presence of Et_3_N). Thus, we modified the synthetic strategy using an alternative route *via* the amino alcohol as follows: reduction of **74** with DIBAH followed by silylation of the resulting alcohol with TBSCl gave the bis-silylated compound **76**, and a coupling reaction with pyrroloiminoquinone **25** yielded **77**. The spirodienone formation of **77** using PIFA effectively proceeded in the presence of MK10 to give a diastereomeric mixture of **78** and **78**′. Both diastereomers were readily separated by column chromatography on silica gel to give the less polar isomer **78** and the polar isomer **78**′ (Scheme 19).

**Scheme 20:**
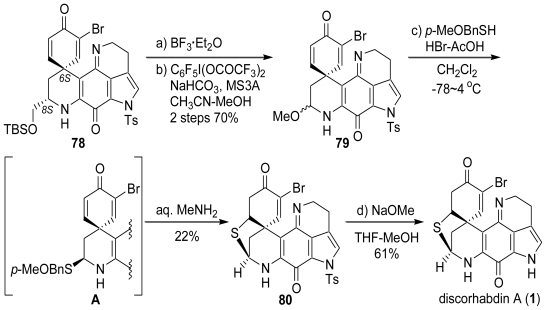
The total synthesis of (+)-discorhabdin A.

The major isomer **78**, whose absolute stereochemistry (*S* configuration) of the spirocenter (C-6) is the same as that of natural discorhabdin A (**1**), was desilylated by BF_3_·Et_2_O, and then converted into the *N,O*-acetal intermediate **79** by an oxidative fragmentation reaction with C_6_F_5_I(OCOCF_3_)_2_ and MeOH. The *p*-methoxybenzylthio group was efficiently introduced in the presence of BF_3_·Et_2_O to give the unstable *N,S*-acetal **A** as a diastereomeric mixture. The debenzylation of **A**, a labile and highly functionalized compound, also required the mildest possible reaction conditions. Our initial strategy was to perform a mild debenzylation on the *p*-methoxybenzylsulfonium salt formed by the 1,4-addition of a sulfide group to the cyclohexadienone moiety. Accordingly, we treated a diastereomeric mixture of **A** with 30% HBr-AcOH followed by aqueous work up with MeNH_2_ that produced the *N*-tosylated discorhabdin A (**80**). Ultimately, we developed an efficient one-pot transformation procedure yielding **80** from **79**. The procedure using *p*-methoxybenzylthiol in 30% HBr-AcOH (36 h; −78~4 °C) followed by treatment with aqueous MeNH_2_ mainly gave **80**: the enantiomeric excess (ee) of **80** was confirmed to be >99% by HPLC analysis using a chiral column (DAICEL CHIRALCEL OD; *n-*Hex/*i-*PrOH = 65/35; 20 °C). Finally, removal of the tosyl group of **80** with NaOMe in THF gave discorhabdin A (**1**) in the optically pure form for the first time (Scheme 20. The synthetic product as its HCl salt was identical in all respects with the natural discorhabdin A including its optical rotation {synthetic discorhabdin A: [α]_D_ +388 (c = 0.06, MeOH); natural discorhabdin A: [α]_D_ +400 (c = 0.05, MeOH)} and ^1^H NMR nOe data.

Furthermore, we also completed the total synthesis of the unnatural discorhabdin A, (−)-discorhabdin A, starting from the d-tyrosine methyl ester by the same route as described for (+)-discorhabdin A (**1**) {synthetic product: [α]_D_ -390 (c = 0.05, MeOH)}.

## 5. Semi-Synthesis of Discorhabdins P and U [57]

**Scheme 21:**
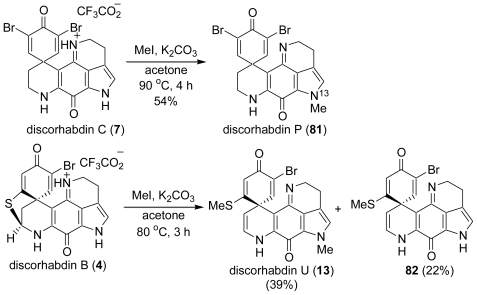
The semi-synthesis of discorhabdin P and U.

In 2006, Copp *et al*. reported the semi-synthetic preparation of discorhabdins P (**81**) and U (**13**) from natural discorhabdins C (**7**) and B (**4**). In New Zealand waters, *Latrunculia* sp. sponges are a rich source of the discorhabdin alkaloids, with organisms capable of producing either discorhabdins A (**1**), B (**4**) or C (**7**) as their major secondary metabolites, typically in isolated yields of 25–35 mg/g dry sponge weight [58]. The aim of the Copp *et al*. study was to investigate the methylation of discorhabdin C (**7**) in an effort to prepare discorhabdin P (**81**) or related *N*-methyl analogs for further biological testing, and for the first time, to prepare similar analogs of discorhabdin B (**4**) in an effort to expand the structure-activity relationship understanding of this class of compounds. Discorhabdin C (**7**) was reacted with CH_3_I in dry acetone to yield a single major product, the 13-*N*-methyl analog discorhabdin P (**81**) in 54% yield (Scheme 21). Discorhabdin B (**4**) was reacted with CH_3_I in dry acetone to yield two products, discorhabdin U (**13**) and **82**. The products of this reaction were dependent upon the molar equivalents of CH_3_I used: with a large excess (10–25 equivalent) yielding predominantly discorhabdin U (**13**), while a two molar equivalent of CH_3_I yielded the mono-methyl **82**. The order of the methylation of discorhabdin B (**4**) preferentially favors the thio group and a large excess of CH_3_I enables the methylation at the *N*-13 pyrrole position.

## 6. Total Synthesis of Prianosin B [44]

**Scheme 22:**
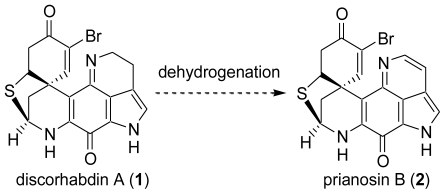
Synthetic strategy for prianosin B.

Although, some 20 years ago, Kobayashi *et al.* also reported one such family, prianosin B (**2**), which has the 16,17-dehydropyrroloiminoquinone structure, its synthesis has not yet been reported [17]. For the synthesis of prianosin B (**2**), the construction of the 16,17-dehydropyrroloiminoquinone structure is an important issue. We have succeeded in the first total synthesis of the sulfur-containing discorhabdin, discorhabdin A [40–43]. We postulated that the prianosins and discorhabdins with the 16,17-dehydropyrroloiminoquinone moiety can be synthesized by dehydrogenation of the pyrroloiminoquinone of the discorhabdin A (**1**) or discorhabdin A intermediate (Scheme 22).

**Scheme 23:**
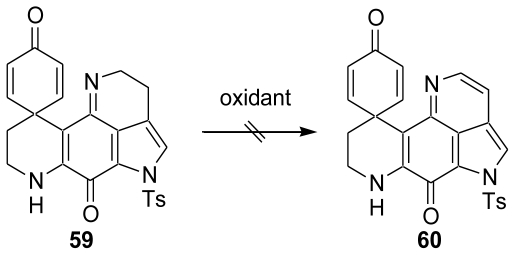
Model study using various oxidants.

We first examined the dehydrogenation reaction of the simple spirodienone (**83**) with various oxidants as the model reaction. However, reactions with oxidants, such as DDQ, CAN, MnO_2_ and Pd/C (Yamamura’s conditions) [59], gave poor results (Scheme 23).

**Scheme 24:**
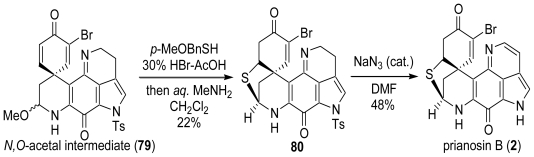
The total synthesis of prianosin B.

We next tried the use of nucleophiles, because White *et al*. reported that the dehydrogenation and detosylation of the pyrroloiminoquinone system using sodium azide as a nucleophile [60]. From a detailed study of nucleophiles, we found that the use of a catalytic amount of NaN_3_ produced the best result. Since it was revealed that the detosylation and dehydrogenation reaction of the pyrroloiminoquinone unit with a catalytic amount of NaN_3_ proceeded in good yield, the total synthesis of prianosin B (**2**) was next studied (Scheme 24). In our own total synthesis of discorhabdin A (**1**), the *N,O*-acetal intermediate (**79**) was prepared from the l-tyrosine methyl ester hydrochloride in eight steps. The sulfur cross linkage reaction of the intermediate was carried out using *p*-MeOBnSH and 30% HBr-AcOH followed by aqueous MeNH_2_ to give the sulfur-linked pyrroloiminoquinone compound **80**. The treatment of **80** with NaN_3_ in DMF caused detosylation and dehydrogenation to produce prianosin B (**2**) in 48% yield. The spectral data were identical to that reported for the natural prianosin B (**2**) ([α]^23^_D_ +362 (c 0.405, CHCl_3_), lit. [α]^30^_D_ +360 (c 0.1, CHCl_3_) [17]).

**Scheme 25:**
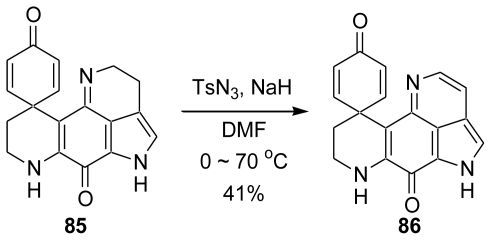
The reaction of *N*-H spirodienone, TsN_3_ and NaH.

For determining the reaction mechanism, two studies were carried out. One was the measurement of the mass spectrum of the reaction mixture. A suspension of spirodienone **83** and NaN_3_ in DMF was stirred under nitrogen at 70 °C for 40 min. The mass spectrum of the reaction mixture revealed the ion peak of TsN_3_. The other was the reaction of the *N*-H spirodienone **85** and TsN_3_ under basic conditions. Thus, NaH and TsN_3_ were added to a solution of **85** in DMF and the aromatic compound **86** was obtained in 41% yield (Scheme 25).

**Scheme 26:**
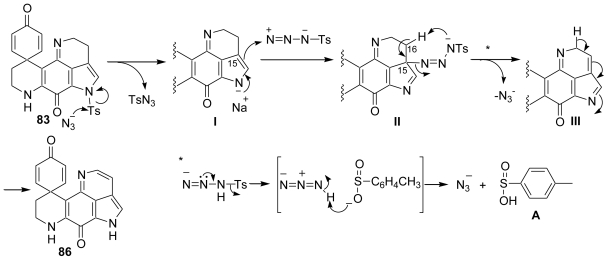
A plausible mechanism for the dehydrogenation reaction of pyrroloiminoquinone using NaN_3_.

A plausible reaction mechanism for the present dehydrogenation reaction of pyrroloiminoquinone using NaN_3_ is illustrated in Scheme 26. It would involve the nucleophilic attack of N_3_^−^ on the tosyl residue followed by the addition of metalo enamine to TsN_3_ to produce the intermediate **I** [61–63]. Dehydrogenation would proceed by the intramolecular elimination *via* a six-membered transition state to produce the intermediate **III** and isomerization to produce **86** [64–65]. Reproduction of the azide anion during the reaction would make possible the use of the catalytic amount of NaN_3_. The reproduction of the azide anion was deduced from the presence of toluenesulfinic acid observed in the ^1^H-NMR spectrum (Scheme 26).

## 7. Summary

In this review, we described the existing studies on the total synthesis of the discorhabdins. Because of their prominent potent antitumor activity and unusual ring structure, synthesis of the discorhabdins has attracted the interests of many organic chemists. Many synthetic approaches have appeared over the past decade, however, only few discorhabdins have been completely synthesized. We have accomplished the total syntheses of discorhabdins C, A and prianosin B. Discorhabdin A has the strongest activity *in vitro*, but no activity *in vivo*. Therefore, we are synthesizing a variety of discorhabdin analogs [66]. Although many synthetic studies of the discorhabdin alkaloids have already appeared, biological studies including structure activity relationship or the mode of action of the discorhabdins have not yet significantly occurred [67–71]. In the next stage, it is necessary to study the structure activity relationship and the mode of action of the discorhabdins.

## Figures and Tables

**Scheme 7:**
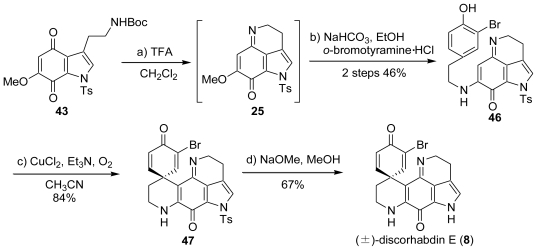
The total synthesis of (±)-discorhabdin E by Heathcock *et al*.
